# Lymphoma of the Urinary Bladder

**DOI:** 10.1155/2014/327917

**Published:** 2014-01-09

**Authors:** Anthony Kodzo-Grey Venyo

**Affiliations:** North Manchester General Hospital, Department of Urology, Delaunays Road Crumpsall, Manchester, UK

## Abstract

*Background*. Lymphoma of the urinary bladder (LUB) is rare. *Aims*. To review the literature on LUB. *Methods*. Various internet databases were used. *Results*. LUB can be either primary or secondary. The tumour has female predominance; most cases occur in middle-age women. Secondary LUB occurs in 10% to 25% of leukemias/lymphomas and in advanced-stage systemic lymphoma. Less than 100 cases have been reported. MALT typically affects adults older than 60 years; 75% are female. Diffuse large B-cell lymphoma is also common and may arise from transformation of MALT. LUB presents with haematuria, dysuria, urinary frequency, nocturia, and abdominal or back pain. Macroscopic examination of LUBs show large discrete tumours centred in the dome or lateral walls of the bladder. Positive staining of LUB varies by the subtype of lymphoma; B-cell lymphomas are CD20 positive. MALT lymphoma is positively stained for CD20, CD19, and FMC7 and negatively stained for CD5, CD10, and CD11c. LUB stains negatively with Pan-keratin, vimentin, CK20, and CK7. MALT lymphoma exhibits t(11; 18)(q21: 21). Radiotherapy is an effective treatment for the MALT type of LUB with no recurrence. *Conclusions.* LUB is diagnosed by its characteristic morphology and immunohistochemical characteristics. Radiotherapy is a useful treatment.

## 1. Introduction

Lymphoma of the urinary bladder is an uncommon lesion; and its diagnostic features may not be well known by the unaccustomed practitioner. The ensuing document contains a review of the literature on lymphoma of the urinary bladder.

## 2. Methods

The key words used for the search were Lymphoma of bladder; lymphoma of urinary bladder; vesical lymphoma. Documentations from 46 sources were found which had discussed various aspects relevant to lymphoma of the urinary bladder and information from these 46 sources were used to write the literature review.

## 3. Literature Review

### 3.1. Overview

*Definition*. Lymphoma of the urinary bladder can be either (a) primary lymphoma of the urinary bladder and this is a rare lymphoma originating in the urinary bladder with no known lymphoma elsewhere or (b) secondary lymphoma of the urinary bladder and this is much more common, and this secondary lymphoma is associated with a primary lymphoma originating in an extra vesical site [1].

*Epidemiology*. Lymphomas of the urinary bladder have a female predominance, and most cases of lymphoma of the urinary bladder occur in middle-age women [1]. Secondary involvement of the urinary bladder occurs in 10% to 25% of leukemias/lymphomas and they occur in advanced-stage systemic lymphoma [1]. Less than 100 cases of lymphoma of the urinary bladder have been reported so far [1]. MALT is the most common subtype of lymphomas in the urinary bladder and this typically affects adults who are more than 60 years old and 75% are female [1]. It has been reported that diffuse large B-cell lymphoma is also common, and it may arise from transformation of MALT [2].

*Sites*. Lymphoma may involve the urinary bladder and the lower ureteral tract [1].

*Clinical Features*. Lymphoma comprises 5% of nonurothelial tumours of the urothelial tract [1]. Kempton et al. [3] reported long median survival for either primary lymphoma of bladder or lymphoma with initial presentation in the urinary bladder but other coexisting diseases [3]. Recurrent lymphoma in the urinary bladder is associated with widely disseminated disease and poor prognosis [1]. It has been stated that low-grade MALT lymphoma is the most common lymphoma subtype in the urinary bladder; it is much more common as secondary tumour than primary tumour, and a history of chronic cystitis is commonly associated with this type of tumour [4].

*Presentation*. Lymphoma of the urinary bladder presents with visible haematuria, dysuria, urinary frequency, nocturia, and abdominal pain or back pain [1]. 

*Radiological Imaging*. The radiological investigations of lymphoma of the urinary bladder reveal submucosal masses: 70% of cases are solitary masses; 20% of cases are multiple masses; and 10% of cases show diffuse bladder wall thickening [1]. 

*Macroscopic Description*. Macroscopic examination of lymphomas of the urinary bladder shows discrete tumours which are large and centred in the dome or lateral walls of the urinary bladder [1].

*Microscopic Description*. MALT lymphomas exhibit sheets of low-grade, uniform cells which surround and separate but do not destroy muscle fascicles [1]. 

*Cytology Description*. MALT lymphomas of the urinary bladder tend to exhibit monomorphic small- to medium-sized lymphocytes [1].

*Immunohistological Staining. *Positive staining of lymphomas of the urinary bladder varies by the subtype of lymphoma; B-cell lymphomas are CD20 positive [1]. 

MALT lymphoma is positively stained for CD20, CD19, and it is negatively stained for CD5, CD10, and CD11c but it is positively stained for FMC7 [1].

Lymphomas of the urinary bladder stain negatively with Pan-keratin, vimentin, CK20, and CK7 [1]. 

*Molecular/Cytogenetics  Description*.  MALT  lymphoma exhibits t(11; 18)(q21 : 21) [1]. 

*Prognostic Factors*. The prognostic factors of lymphoma of the urinary bladder include histological subtype and the stage of the tumour [1].

*Treatment*. Radiotherapy is the treatment for the MALT type of lymphoma of the urinary bladder and usually there is no recurrence of tumour following such treatment [1]. 

*Differential Diagnosis*. The differential diagnoses of lymphoma of the urinary bladder include

- urothelial carcinoma with prominent lymphoid infiltrate [5];
- undifferentiated carcinoma [1].

### 3.2. Narrations from Reported Cases

Cohen et al. [6] stated that the first recorded case of lymphoma of the bladder was reported by Eve and Chaffey in 1885 [6]. They also stated that malignant lymphoma of the urinary bladder can be classified into one of three different clinical groups as follows:

- primary lymphoma localized to the bladder;
- lymphoma presenting in the bladder as the first sign of disseminated disease (nonlocalized lymphoma);
- recurrent urinary bladder involvement by lymphoma in patients with a history of malignant lymphoma (secondary lymphoma).

Cohen et al. [6] also stated that primary extranodal marginal zone lymphoma of mucosa-associated lymphoid tissue (MALT type) of the urinary bladder, which was first described by Kempton et al. [3] in 1990, is the most common primary bladder lymphoma and is associated with an excellent prognosis. Cohen et al. [6] also reported a patient with visible haematuria who was found to have a primary lymphoma of the urinary bladder.

Bates et al. [2] reported the clinical and histological features and outcomes of primary and secondary malignant lymphomas of the urinary bladder [2]. They obtained eleven cases of malignant lymphoma of the urinary bladder from the registry of cases of St Bartholomew's and the Royal London Hospitals in the UK. They classified the lymphomas on the basis of their morphology immunophenotype. They also reviewed the clinical records of the patients. Bates et al. [2] reported that there were six primary lymphomas: three extranodal marginal zone lymphomas of mucosa-associated lymphoid tissue (MALT) type and three diffuse large B-cell lymphomas. Of the five secondary cases, four were diffuse large B-cell lymphomas, one secondary to a systemic follicular centre lymphoma, and one nodular sclerosis Hodgkin's disease. Bates et al. [2] also reported the following.

- Four patients with secondary lymphoma, for whom followup was available, had died of disease within 13 months of diagnosis.
- Primary lymphomas followed a more indolent course.
- In one case, there was evidence of transformation from low-grade MALT type to diffuse B-cell lymphoma.
- The most common presenting symptom was haematuria.
- The cystoscopic appearances were of solid, sometimes necrotic, tumours which resembled transitional cell carcinomas, and in one case the tumours were multiple.

Bates et al. [2] stated that these cases represented 0.2% of all neoplasms of the urinary bladder. Bates et al. [2] made the following conclusions.

- Diffuse large B-cell lymphoma and MALT-type lymphoma are the most common primary malignant lymphomas of the bladder.
- Lymphoepithelial lesions in MALT-type lymphoma involve transitional epithelium, and their presence in high-grade lymphoma suggests a primary origin owing to transformation of low-grade MALT-type lymphoma.
- Primary and secondary diffuse large B-cell lymphomas of the bladder are histologically similar, but the prognosis of the former is favourable.

Kempton et al. [3] studied patients with malignant lymphoma of the bladder, and they defined three clinical groups: those with primary lymphoma localized in the bladder, lymphoma presenting in the bladder as the first sign of disseminated disease (nonlocalized lymphoma), and recurrent bladder involvement by lymphoma in patients with a history of malignant lymphoma (secondary lymphoma). They studied differences in these groups regarding lymphoma type, clinical presentation, and clinical outcome. Kempton et al. [3] searched the Mayo Clinic Tissue Registry records from 1940 to 1996 to identify patients with lymphomas involving the bladder. The lymphomas were classified based on review of the histology and immunophenotype performed by immunoperoxidase methods. Kempton et al. [3] also reviewed the clinical records. They reported the following.

- The presenting symptoms included urinary frequency, dysuria, haematuria, and lower abdominal and back pain.
- Primary lymphoma was present in six patients. All were B-cell-lineage, low-grade lymphomas of the mucosa-associated lymphoid tissue (MALT) type.
- No patient had recurrent lymphoma or died of lymphoma.
- Nonlocalized bladder lymphoma occurred in 17 patients: one with low-grade lymphoma of the MALT type, four with follicle centre lymphomas, and 12 with large cell lymphomas.
- Excluding two patients who died postoperatively, median survival was 9 years. Six patients died of lymphoma in the follow-up period.
- Secondary bladder lymphoma occurred in 13 patients: two with low-grade lymphoma of the MALT type, one with follicle centre lymphoma, one with mantle cell lymphoma, and nine with diffuse large cell lymphomas. Median survival in this group was 0.6 years.
- Low-grade lymphoma of the MALT type was the most frequent type of primary bladder lymphoma and was associated with an excellent prognosis.

They concluded that:

- The bladder can be the presenting site of lymphomatous involvement in patients with more widespread disease.
- Survival in this group is quite favourable and is presumably dependent on lymphoma histologic type, stage of disease, and other prognostic factors.
- Bladder involvement by recurrent lymphoma is a sign of widely disseminated disease and is associated with a very poor prognosis.

Al-Maghrabi et al. [4] in 2001 stated that primary lymphoma of the urinary bladder is rare and only 84 cases were reported in the English literature at the time of their publication, but none of these cases had had molecular confirmation of clonal immunoglobulin gene rearrangement. They reviewed all cases with primary urinary bladder lymphoma in their records to classify them using the REAL classification, to confirm their immunophenotype and genotype, and to determine their outcome. They identified 4 cases of primary urinary bladder lymphoma in their medical records from a 30-year period. They performed immunohistochemical detection of immunoglobulin light chains and molecular analysis of immunoglobulin heavy-chain genes using the polymerase chain reaction on paraffin-embedded material. They reported the following.

- All patients were older than 60 years.
- The male-female ratio was 1 : 3.
- All patients had a history of chronic cystitis.
- Histologic features of mucosa-associated lymphoid tissue lymphoma with centrocyte-like cells, plasmacytoid occurred.
- B cells or both were observed in all cases.
- Monoclonality of B cells was demonstrated by immunohistochemistry, polymerase chain reaction, or both methods in every case.
- All patients presented with stage IAE disease were treated with radiotherapy alone and had been in continuous complete remission for 2 to 13 years.
- On immunophenotyping, light-chain restriction was demonstrated in 3 cases (cases 2, 3, and 4) (results are summarized in Table 2).
- Flow cytometric data were available for case 4 and showed typical marginal zone B-cell immunophenotype (positive for CD45 and CD20; negative for CD5, CD23, and CD10) with k light-chain restriction and an S phase of 1%, which is consistent with a low-grade lymphoma.
- PCR for immunoglobulin heavy-chain gene polymerase chain reaction analyses (Figure 3) revealed clonal immunoglobulin heavy-chain (IgH) gene rearrangement in 3 cases (cases 1, 2, and 4); PCR was not informative in case 3. Immunohistochemistry, however, showed k light-chain restriction as well as a heavy-chain restriction in this case.

They concluded the following.

**Figure 1:**
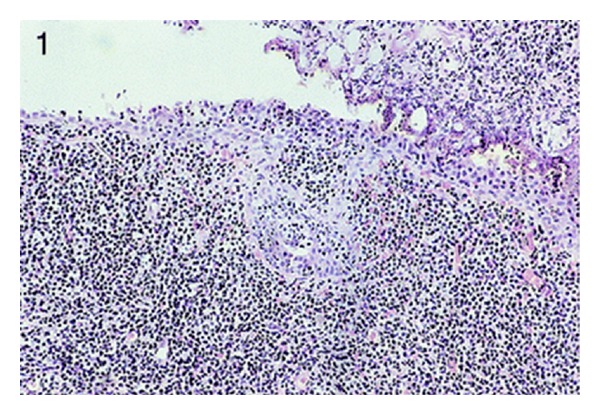
The microscopic feature of the second cases (haematoxylin and eosin staining). Case 2: mucosa-associated lymphoid tissue lymphoma involving the lamina propria of the urinary bladder (hematoxylin-eosin, original magnification ×100). Reproduced from [4] with permission of the Editor-in-Chief of Archives of Pathology and Laboratory Medicine on behalf of the editorial team of the journal and the American Association of Pathology.

**Figure 2:**
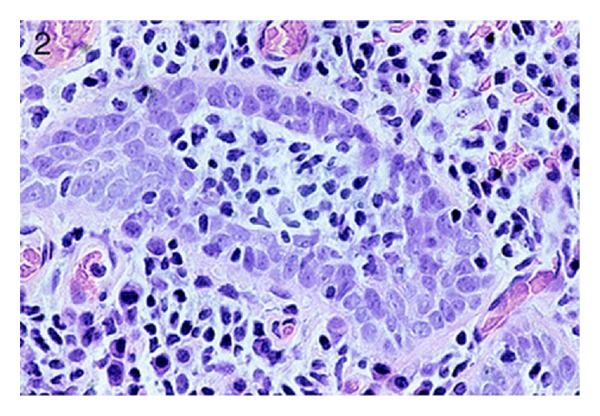
The microscopic feature of the second cases (haematoxylin and eosin staining). Case 2: focal lymphoepithelial lesions in area of cystitis glandularis (hematoxylin-eosin, original magnification ×400). Reproduced from [4] with permission of the Editor-in-Chief of Archives of Pathology and Laboratory Medicine on behalf of the editorial team of the journal and the American Association of Pathology.

**Figure 3:**
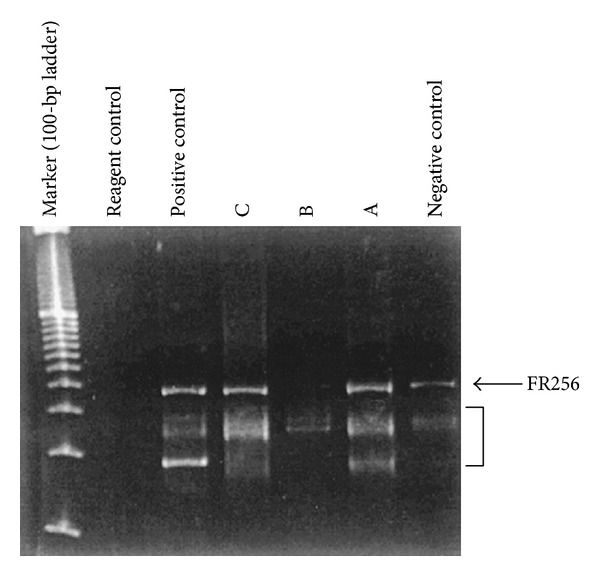
Case 4: B-cell-specific polymerase chain reaction using primers directed at the framework 256 (FR256) regions of the immunoglobulin heavy-chain gene (IgH). The top arrow represents the internal control that is used to ensure the presence of amplifiable DNAin each sample. The bracket in the FR256 figure denotes the size range in which IgH gene products can be seen. Although the DNA is degraded and the signal is weak, patient B (case 4) clearly shows the presence of a clonally rearranged IgH gene using the FR256 primers. Clonal rearrangements of IgH genes were also noted in cases 1 and 2 (not shown in figure). Lanes A and C are from cases unrelated to this paper. Reproduced from [4] with permission of the Editor-in-Chief of Archives of Pathology and Laboratory Medicine on behalf of the editorial team of the journal and the American Association of Pathology.

- Primary bladder lymphomas are usually of low-grade mucosa-associated lymphoid tissue type.
- They were more common in females and had been associated with a history of chronic cystitis.
- Lymphoepithelial lesions were seen only in association with areas of cystitis glandularis.
- B-cell clonality was readily demonstrable by immunohistochemistry and/or polymerase chain reaction analysis.
- Local radiotherapy appeared to confer long-term control (see Figures 1, 2, and 3 which show the morphology of 3 of the four tumours and Table 1 which shows the clinical findings of the 4 cases and Table 2 which shows the immunohistochemical and polymerase chain reaction (PCR) findings of the tumours).

Sufrin et al. [7] reported on a study of 599 patients who had died of malignant lymphoma between 1952 and 1972 and this revealed involvement of the bladder in 13 per cent. Bladder involvement was always a secondary event, which occurred in association with disseminated disease and was more common in non-Hodgkin's lymphoma than in Hodgkin's disease. They reported the following.

- Direct infiltration from adjacent pelvic foci as well as discrete apparent metastatic foci was noted.
- Involvement was usually microscopic although the presence of gross disease was invariably clinically manifest.
- Cystoscopy and cystography were valuable in the diagnosis of gross lesions.
- In contrast to primary vesical lymphoma, the treatment of secondary vesical lymphoma was symptomatic and an operation was indicated rarely.
- Local radiotherapy was effective in treating the symptoms of secondary vesical lymphoma.

Kuhara et al. [8] reported a patient with primary malignant lymphoma of the urinary bladder. They reported that grossly, the bladder showed multiple submucosal masses. Histologically and immunohistochemically, diffuse B-cell lymphoma of the medium-sized cell type was revealed. They stated the following.

- On the basis of clinicopathological features, the case resembled previously recorded cases of bladder lymphoma.
- The pathogenesis of the primary bladder lymphoma was presumably associated with follicular or chronic cystitis.
- Primary lymphoma of the bladder is a condition that is very rarely included in a series of extranodal lymphomas, and there is a curious sex difference in its occurrence rates between Japan and Western countries.
- Primary lymphoma of the bladder may be considered a lymphoma that originates from mucosa-associated lymphoid tissue.

Sönmezer et al. [9] reported a 58-year-old woman who was suffering from chronic pelvic pain, pelvic pressure, dysuria, and genitourinary bleeding for 2 months. On gynaecological examination, her uterus was found to have a semifixed cervix which was mobile and a solid, irregular mass was also found with a size of 10 cm that was located anterior to her right adnexa. She had ultrasound scan which confirmed a solid mass with indefinite borders. Her CA 125 level was normal. On digital rectal examination, the rectal mucosa was normal. PAP smear and endometrial biopsy were without any pathology. To find out the origin of the bleeding, a cystoscopy evaluation and an intravenous pyelogram were performed which revealed an irregular, necrotic, solid submucosal mass at the dome of the bladder. Histopathological and immunophenotypic evaluation of the biopsy specimen performed thereafter by immunoperoxidase methods using Streptavidin-Biotin peroxidase system revealed a high-grade B-type malignant lymphoma. Following the establishment of the diagnosis as postmenopausal adnexal mass and lymphoma of the urinary bladder, the patient was reevaluated with clinical investigations which included complete blood count, tumour markers, peripheral blood smear and bone marrow biopsy, and radiological investigations which included computed tomography; these revealed no evidence of tumour elsewhere. An exploratory-laparotomy was undertaken which revealed that the urinary bladder was adherent to the anterior abdominal wall, omentum, uterus, and right adnexa forming a conglomerate mass. The peritoneal surface was free of any metastatic spread. There was no sign of fistula formation. After mobilization of the bladder, a cystotomy was performed and a solid, necrotic mass originating from bladder mucosa at the dome was found which also invaded the bladder wall. The mass was removed completely by partial cystectomy with a 2 cm margin of normal tissue. Total abdominal hysterectomy and bilateral oophorectomy were also performed. The retroperitoneal lymph nodes were found not to be enlarged by palpation. She received four courses of CHOP regimen (cyclophosphamide, vincristine, doxorubucin, and prednisolone). The rebiopsy performed from the dome of the bladder on the 3rd and 6th months postoperatively were normal. During a followup of 6 years, the patient was complaint-free and no local or distant recurrence was found. Sönmezer et al. [9] stated that primary lymphoma of the urinary bladder is a fairly uncommon entity, whereas urinary tract involvement is reported in up to 13% of the cases with advanced systemic disease [7].

Primary malignant lymphoma of the urinary bladder was first described in 1885 and marked female preponderance was reported with a female to male ratio of 6.5 : 1 [10].

The most common types of primary lymphoma of the urinary bladder are

- extranodal marginal zone lymphoma of mucosa-associated lymphoid tissue type (MALT-type lymphoma)
- diffuse large B-cell lymphoma.

The development of lymphomas in a site normally not including lymphoid tissue was explained by the MALT concept, which was first described by Isaacson and Wright. They in 1983 [28] reported a 58-year-old woman who had primary high-grade B-type malignant lymphoma and who presented with genitourinary bleeding and a large pelvic mass that appeared as a gynaecological tumour [28].

Ohsawa et al. [10] stated the following.

- In mucosa-associated lymphoid tissue, such as neoplasms arising in indigenous lymphoid tissues, primary malignant lymphoma of the urinary bladder is a fairly uncommon disease and this accounts for 0.2% of cases with extranodal lymphoma, mostly appearing in the sixth decade.
- It had been stated that a significant proportion of extranodal non-Hodgkin lymphomas is known to arise from the intestine or lung, or from chronic inflammatory conditions. Since lymphoid tissue is normally not found in the urinary bladder, preexisting chronic inflammation is postulated as the origin. Nevertheless, in most of the cases, as in their case, history of chronic cystitis and histologic evidence of such an inflammation were lacking [2, 10, 27] and therefore uncertainty still exists regarding the role of chronic cystitis in the development of lymphoma.
- A review of the literature revealed that the most apparent symptoms of lymphoma of the urinary bladder are haematuria, dysuria, nocturia, urinary frequency, suprapubic or abdominal pain, weight loss, and anorexia [10, 27].

Isaacson and Wright [28] stated the following.

- In their reported case, the disease (lymphoma of the bladder) presented as a pelvic mass showing strict adhesions with adjacent pelvis organs which resembled a genital cancer. But they initially thought that the patient had both a primary lymphoma of the bladder and an adnexal mass.
- Following an extensive work-up and histopathological evaluation of the biopsy specimen, a high-grade B-type lymphoma was diagnosed.

Lymphoma of the bladder is proposed to have characteristic cystoscopic appearance that can aid in diagnosis and is usually described as a smooth, nonulcerative, friable, or haemorrhagic submucosal tumour [26].

It was stated that treatment of patients with primary lymphoma of the bladder includes many options with favourable prognosis. Ohsawa et al. [10] stated that:

- In their review of the literature, they found that multimodality therapy including surgical resection followed by chemotherapy or radiation therapy provided favourable prognosis.
- Only 3 of 27 patients died and 23 of 24 patients had no evidence of disease at the 31-month followup.
- In another review by Kempton et al. [3], none of the 6 patients died as a result of lymphoma.
- The extent of surgery did not seem to affect the prognosis, since a similar proportion died or had recurrence, regardless of total or partial cystectomy/resection performed [27].
- The combination therapy including surgery and chemotherapy resulted in a 6-year disease-free survival in their patient.
- Based on the findings in the literature and in their case, they concluded that therapy including surgery along with radiation or chemotherapy for primary lymphoma of the bladder provides a good prognosis, even in case of a large adhesive mass.

Ando et al. [22] reported a 77-year-old woman, who presented with a sensation of urinary retention and symptoms which were suggestive of cystitis and she was treated with antibiotics, but her symptoms did not subside. She had an intravenous pyelogram and cystoscopy which revealed a wide-based submucosal mass which measured 3 cm in the left wall of the urinary bladder. Histological findings of the tissue which was obtained by means of transurethral resection (TUR) revealed a dense, monomorphic atypical lymphoid (centrocyte-like) infiltrate with reactive lymph follicles in the subepithelial tissue. Monocytoid and plasmacytoid features were readily evident in a population of these cells. Lymphoepithelial lesions involving the urothelium were also noticed in some areas. These features were considered to be strongly suggestive of primary low-grade B-cell lymphoma of the MALT type. The diagnosis was confirmed by immunohistochemical and flow cytometric studies, both of which showed a clear immunoglobulin restriction to lambda light chain and also by polymerase chain reaction-based assay using a formalin-fixed paraffin-embedded TUR tissue sample, which showed a clonal *Ig *heavy-chain gene rearrangement. Clinical staging procedures revealed that the tumour was localized in the urinary bladder. The patient did not receive any chemotherapy and she was alive and well with no evidence of recurrence, 3 years after she had undergone trans-urethral resection (TUR) of her bladder tumour. Ando et al. [22] stated that the case demonstrated that these ancillary tests are worth-performing for the confirmation of B-cell clonality in trans-urethral resected (TUR) tissue samples showing dense B-Iymphocytic infiltration.

Tsiriopoulos et al. [25] reported the case of a 76-year-old woman who had a past medical history of low-grade chronic lymphocytic leukaemia. She presented with severe chronic bladder symptoms which were attributed to interstitial cystitis. She underwent cystectomy and ileal conduit formation after the failure of all conventional treatments. Histopathological examination of the bladder revealed primary splenic marginal zone lymphoma. They reviewed the literature which showed the rarity of such nonhematopoietic visceral metastases. They stated that their case may represent the first reported splenic marginal zone lymphoma with bladder involvement and highlighted the clinical and histological similarities with interstitial cystitis.

Rijo et al. [35] reported a 27-year-old female who presented with acute urinary retention. She underwent gynaecological examination which revealed a 30 mm × 40 mm × 30 mm widely pedunculated, firm, smooth, paraurethral mass without discharge, arising close to the external urethral orifice. Her past medical and surgical history was otherwise unremarkable, with no history of previous urinary tract symptoms. She had voiding cystourethrogram (VCUG) and computed tomography (CT) scan which revealed a paraurethral mass. Pelvic magnetic resonance imaging (MRI) was performed as a supplementary diagnostic tool and this confirmed the presence of a large, well-circumscribed, paraurethral mass. She underwent cystoscopy which confirmed the urethral protrusions at the bladder neck region. A provisional diagnosis of paraurethral leiomyoma was made on the basis of the cystoscopic examination, as well as radiological and clinical findings. The proposed treatment involved surgical removal of the mass. An open vaginal approach was selected; the paraurethral tissue was diffusely infiltrated and the mass was partially removed. Intraoperative frozen section histological examination showed a small-cell lymphoproliferative tumour, so the surgical procedure was discontinued. The postoperative course was uneventful, and the urethral catheter was left inside the urinary bladder for three weeks. After removal of the urethral catheter, the patient developed mild stress urinary incontinence.

Histology of the  haematoxylin-eosin-stained tissue revealed a highly cellularized tumour which displayed a diffuse, infiltrating pattern, a medullary, cohesive proliferation of medium-sized neoplastic cells, monomorphic, medium-sized cells with round nuclei, multiple nucleoli, and a basophilic cytoplasm. A “starry-sky” pattern was observed with frequent mitotic figures.

Immunohistochemical stains were negative for antibodies against CD23, CD3, CD5, bcl2, bcl6, TDT, and p53. Tumour cells were positive for CD79a, CD20, CD43, CD10, MUM1, and Ki67 (100%). Fluorescence in situ hybridization (FISH) for MYC/IGH/CEP8 revealed t(8; 14)(q24; q32). However, Epstein-Barr virus RNA was not detectable. Polymerase chain reaction (PCR) analysis was used to analyse the rearrangement of VH region genes. By amplifying the complementarity-determining region III using PCR, it was discovered that CDRIII, CDRII, and CDRI showed a clonal pattern. All of the phenotypic features mentioned supported the diagnosis of Burkitt's lymphoma.

Based on the presumptive diagnosis of primary paraurethral Burkitt lymphoma (BL), the patient had a full workup that included a bone marrow aspirate/biopsy, viral serologies, MRI evaluation, and PET/CT to rule out metastatic origin of the paraurethral Burkitt's lymphoma (BL). The bone marrow aspirate and biopsy revealed normocellular haematopoiesis, and no tumour cells were detected based on negative immunohistochemical analysis (CD79a, CD20, and CD3). Tumour markers and a screening test for Epstein-Barr virus, human immunodeficiency virus, hepatitis virus, and cytomegalovirus were all negative. She had magnetic resonance imaging (MRI) scan which showed a T2-weighted hypersignal at the fifth lumbar vertebra. The F-2-fluoro-D-deoxyglucose positron emission tomography CT (FDG-PET/CT) revealed increased FDG uptake in pelvic, bilateral iliac internal/external lymph nodes, and significant activity in the fifth lumbar vertebra.

The patient was referred for six cycles of immunochemotherapy: anti-CD20 (Rituximab) combined with chemotherapy (high doses of methotrexate and cytarabine with conventional cystostatics and prophylactic administration of G-CSF after chemotherapy cycles).

After the completion of the third cycle of treatment, the patient achieved near-complete remission as well as a nearly complete regression of the paraurethral tumor and the lesion of the 5th lumbar vertebra. Haematological grade 2 toxicity and gastrointestinal grade 1 toxicity were reported.

Her followup was uneventful, and at the nine-month followup a total body computed tomography (CT) scan revealed no evidence of clinical progression (either local recurrence or other distant metastasis).

The patient was still alive with a good quality of life and without clinical evidence of tumour progression.

Some authors [23, 24] stated that primary genitourinary lymphomas are uncommon, and, in particular, primary Burkitt's lymphoma (BL) of the bladder or genitourinary tissue is extremely rare [23, 37].

Other authors stated that most frequently, genitourinary lymphoma reflects widespread metastasis which was caused by a systemic haematological disease [37].

Burkitt's lymphoma (BL) was first described in 1958 in Uganda by a surgeon who observed children with rapidly enlarging tumours which involved the jaw. Since then, Burkitt's lymphoma (BL) has been categorized by the World Health Organization (WHO) into three types which include the endemic type, the sporadic type, and the immunodeficiency-associated types [38].

It has been stated that the endemic form of Burkitt's lymphoma is found mostly in equatorial Africa and in Papua New Guinea and this form of Burkitt's lymphoma is associated with the Epstein-Barr virus in 95% of cases. The sporadic (or American) form of Burkitt's lymphoma is found in North America, Northern and Eastern Europe, and the Far East and this form of Burkitt's lymphoma is associated with the Epstein-Barr virus in 15% of patients. The immunodeficiency associated form of Burkitt's lymphoma occurs mainly in patients with HIV, but it can also occur in allograft recipients and patients with congenital immunodeficiencies or X-linked lymphoproliferative disease [38, 39].

It was also stated that:

- Even though Burkitt's lymphoma can involve the head and neck in children, the gastrointestinal tract, genitourinary tract, gonads, mesentery, peritoneum, and retroperitoneum also represent potentially affected sites.
- Lymphomas arising in the male genitourinary tract are relatively uncommon.
- Malignant lymphoma involving the prostate is rare and accounts for less than 0.1% of newly diagnosed lymphomas.

The most frequent presentation forms are obstructive urinary symptoms. [40–42].

Some authors [43, 44] stated the following.

- Bladder outlet obstruction in women is an infrequently diagnosed urological condition.
- A combination of history taking; physical examination; and diagnostic tests provides a consistent way to accurately recognize and diagnose bladder outlet obstruction.

The causes of obstruction are varied and numerous but generally fall within two broad categories: functional and anatomic. In a fertile female, the most likely anatomic causes of bladder outlet obstruction symptoms are bladder and urethral leiomyoma, and an association with female hormone expression has been suggested previously [9, 43, 44].

Other differential diagnoses include urethral caruncle, urethral diverticulum [45, 46], malignant lymphoma, sarcoma, extravesical leiomyoma of the bladder, Gartner's duct cyst, and ectopic urethral orifice [35].

Rijo et al. [35] stated the following.

- The diagnosis of Burkitt's lymphoma depends upon morphological findings, immunophenotyping results, and cytogenetic features. Because this lymphoma is one of the most rapidly proliferating neoplasms and is often associated with tumour lysis syndrome, a prompt diagnosis is required.
- Treatment of Burkitt's lymphoma is inclusive of high doses of alkylating agents, frequent administration of chemotherapy, and attention to central nervous system (CNS) prophylaxis with high doses of systemic chemotherapy, intrathecal therapy, or both.
- There is no role for radiation therapy in the modern treatment of Burkitt's lymphoma—even for localized disease or para—spinal presentations, which respond very quickly to chemotherapy.
- To their knowledge, their reported case of female paraurethral Burkitt's lymphoma was the first case of primary paraurethral female Burkitt's lymphoma not related to Epstein-Barr virus which was reported in the literature.

Thomas et al. [46] stated that intensive chemotherapy regimens are required to treat Burkitt's lymphoma. Although several reports utilized initial excision, radiotherapy, chemotherapy, or some combination thereof, their case report suggested that the use of intensive immunochemotherapy should be considered as a possible treatment modality.

Mourad et al. [21] stated that less than 100 cases of primary lymphoma of the urinary bladder had been reported and most of them were B-cell lymphoma. They reported a case of primary T-cell lymphoma of the urinary bladder in a patient with a history of schistosomiasis. They reported a 52-year-old man who presented with suprapubic discomfort and haematuria. On examination, he was found to have a suprapubic mass. He had computed tomography scan of the pelvis which showed a large lobular mass that occupied the urinary bladder. There was no evidence of any pelvic or abdominal lymphadenopathy and the results of metastatic work-up were negative. The patient underwent a trans-urethral biopsy of the bladder mass and histological examination of the biopsy revealed a diffuse large cell lymphoma which was negative for the B-cell marker L-26 (CD20) and positive for the T-cell marker CD-3. Mourad et al. [21] reported that polymerase chain reaction studies of the paraffin-embedded tissue revealed rearrangement of the T-cell receptor gamma gene. The patient was treated by means of CHOPP chemotherapy and he received cyclophosphamide, doxorubicin, vincristine, and prednisone. Mourad et al. [21] stated that their case, represented to their knowledge, a very rare primary lymphoproliferative neoplasm of the urinary bladder that might represent an unusual immune response to schistosomiasis [21].

Wang et al. [20] stated that primary bladder lymphoma, a rare form of non-Hodgkin's lymphoma that is confined to the urinary bladder, is usually of B-cell origin. They reported an extremely rare case of primary T-cell lymphoma of the urinary bladder. Wang et al. [20] reported a 45-year-old man who presented with haematuria, dysuria, and loin pain. He had ultrasound scan and computed tomography scan which showed a thickened left bladder wall and left hydroureteronephrosis. A diagnosis of primary T-cell lymphoma of the urinary bladder was made which was based upon clinical, radiological, and histological findings. The patient underwent trans-urethral resection of his bladder lesion and following this he was treated with four cycles of CHOPP (cyclophosphamide, doxorubicin, vincristine, and prednisone) chemotherapy. He showed good response and remained in clinical remission 12 months after treatment. [20].

Oh and Zang [19] stated that involvement of the lower urinary tract by advanced non-Hodgkin's lymphoma (HL) had been reported in up to 13% of cases; however, primary non-Hodgkin's lymphoma of the urinary bladder is rare. They reported a 35-year-old man who was admitted with a history of visible haematuria and left flank pain. He underwent cystoscopy which revealed an oedematous broad-based mass on the left lateral wall of the bladder. He had trans-urethral biopsy and histological examination of the specimen revealed non-Hodgkin's lymphoma, diffuse large B-cell type. He had computed tomography scan which revealed left-sided hydronephrosis and hydroureter with left proximal ureter infiltration and thickening of the left lateral wall of the bladder with perivesical fat infiltration without lymph node enlargement. He also had full-scale staging work-up which revealed the bone marrow as the solely involved site. The lesions of the urinary bladder and left urinary tract had completely regressed pursuant to two cycles of systemic cyclophosphamide, doxorubicin, vincristine, and prednisone (CHOPP) chemotherapy with simultaneous restoration of urinary function [19].

Sundaram and Zhang [18] reported an unusual case of localized Epstein-Barr virus (EBV) positive B-cell lymphoproliferative disorder (LPD)/polymorphous B-cell lymphoma of the urinary bladder in a 67-year-old female patient. They reported that the patient had no known predisposing immunodeficiencies and she presented with a recent onset of haematuria. She had a computed tomography scan and cystoscopy which revealed a localized 2.5 cm polypoid or plaque-like mucosal mass on the right posterior and lateral wall of the urinary bladder. Histological examination of the biopsy specimen of the mass showed a diffuse and densely polymorphous atypical lymphoid infiltrate admixed with numerous small lymphocytes, histiocytes, and occasional plasma cells and neutrophils. On immunohistochemical staining, the large atypical cells were positively stained for CD20, CD79a, CD30, and CD43; and they were strongly positive for Epstein-Barr virus (EBV) by in situ hybridization using anti-EBER-1 probe. They also reported that polymerase chain reaction (PCR) for immunoglobulin heavy chain gene rearrangement study showed a clonal gene rearrangement. Sundaram and Zhang [18] made the ensuing statements.

- Primary lymphoma of the bladder is rare and primary Epstein-Barr virus (EBV) + lymphoproliferative disorder (LPD) of the bladder had not been previously described.
- Potential misdiagnosis of poorly differentiated urothelial carcinoma can occur and accurate diagnosis depends upon comprehensive immunohistochemical and molecular work-ups [18].

Abraham Jr. et al. [17] stated that monocytoid B-cell lymphoma (MBCL) is a low-grade neoplasm which is considered to be the neoplastic counterpart to monocytoid B-cell lymphocytes, derived from marginal zone lymphocytes. Abraham Jr. et al. [17] reported a 72-year-old woman who presented with urinary symptoms of burning, urgency, and haematuria. Cystoscopic examination revealed an exophytic mass at the base of the urinary bladder. The lesion was suspected based upon the gross examination findings to be a transitional cell carcinoma; however, on initial histological examination of the biopsied specimen, it was found to be lymphoma which was composed of cells with moderately abundant cytoplasm and an overall size reminiscent of a large-cell type. Following detailed histological examination of the specimen, a diagnosis of monocytoid B-cell lymphoma (MBCL) in the submucosal site was made. She underwent clinical staging which did not show any evidence of lymphoma in any other organs. The patient responded to therapy for non-Hodgkin's lymphoma (NHL). Abraham Jr. et al. [17] stated that their case represented an unusual presentation of low-grade non-Hodgkin's lymphoma (NHL) and it may be consistent with previous suggestions of a relationship between monocytoid B-cell lymphoma (MBCL) and lymphomas of mucosa-associated lymphoid tissue.

Hayashi et al. [16] stated that primary lymphoma of the urinary bladder is quite rare and primarily it is extranodal marginal zone B-cell lymphoma-associated lymphoid tissue (MALT-lymphoma). They stated that prior to their publication there was only one case report of primary diffuse large B-cell lymphoma (DLBCL) of the bladder, accompanied by diffuse wall thickening of the urinary bladder. Hayashi et al. [16] reported the second case of primary DLBCL of the urinary bladder in a 75-year-old woman, who initially presented with acute renal failure. She received three courses of R-CHOPP chemotherapy which were effective to treat her acute renal failure caused by postrenal obstruction and to attain clinical remission.

Siegel and Napoli [15] described a malignant lymphoma which involved the dome and the anterior wall of the urinary bladder in an elderly woman. The initial biopsy showed a malignant neoplasm of uncertain cell type. In view of the fact that the clinical presentation was most compatible with urachal adenocarcinoma, an extensive resection was performed. Microscopic examination revealed that the excised tumour was composed of large lymphoid cells with isolated and clustered signet-ring cells. They reported that immunohistochemical analysis of the tumour established the B-cell phenotype of the neoplasm, and electron microscopy of the signet-ring cells revealed endoplasmic reticulum-bound inclusions consistent with immunoglobulin. Siegel and Napoli [15] stated that primary malignant lymphomas of the urinary bladder are rare, and, to their knowledge, their report was the first example with signet-ring cells. They iterated that the histopathological finding would be a cause of potential confusion with urachal adenocarcinoma.

Kakuta et al. [14] stated that primary mucosa-associated lymphoid tissue (MALT) lymphoma of the bladder is a rare disease, and the most effective therapeutic procedure remains unknown. Kakuta et al. [14] reported a case of primary MALT lymphoma of the bladder which regressed after rituximab in combination with CHOPP chemotherapy (R-CHOOP). The patient was an 84-year-old woman who presented with general fatigue and weight loss. She had a computed tomography (CT) scan which showed a solitary mass in the bladder. She had trans-urethral biopsy of the lesion and histological examination of the specimen revealed extranodal marginal zone B-cell lymphoma of MALT. She had one cycle of R-CHOPP chemotherapy which resulted in her complete remission. They stated that their reported case was the fourteenth case of MALT lymphoma of the bladder in Japan.

Takahara et al. [13] reported an 85-year-old woman who presented with macroscopic haematuria and pain on micturition. She had a cystoscopy which revealed a wide-based submucosal mass, and biopsied specimens of the mass were examined histologically which showed a B-cell lymphoma of the MALT type. She had computed tomography scan which showed a 7.5 cm × 3.0 cm solitary mass lesion situated from the anterior wall to the right lateral bladder wall and magnetic resonance imaging (MRI) scan which showed a low intensity in T1W1, high in T2W1 without invasion. She underwent trans-urethral resection of the lesion. Histological examination of the specimen was consistent with extranodal marginal zone B-cell lymphoma of the MALT type. There was no evidence of lymphoma on computed tomography (CT) of the pelvis, chest X-ray, and Gallium scintigraphy. The patient had stages I (AE) lymphoma. She was treated with radiation therapy (radiotherapy) to the urinary bladder and pelvis (40 Gy in 20 fractions) and she was followed up with computed tomography every 3 months. She had no evidence of recurrence.

Terasaki et al. [12] reported a 64-year-old woman who presented with a history of general malaise. Her haemoglobin level was 9.0 g/dL. She had gastrointestinal endoscopy which revealed a haemorrhagic gastric ulcer, which was considered as aetiology of the anaemia. She had abdominal ultrasound scan which showed bilateral hydronephrosis and hydroureters. Her urine test revealed pyuria and macroscopic haematuria and her urine culture revealed 10(8) colony-forming units of *Escherichia coli* per mL. She had pelvic magnetic resonance imaging which showed thickening of the posterior wall and trigone of the urinary bladder. She underwent trans-urethral resection and biopsy of the mucosa of the urinary bladder which upon histological examination gave a diagnosis of primary mucosa-associated lymphoid tissue (MALT) lymphoma of the urinary bladder. Ann Arbor's clinical stage was IEA. It was planned that she should be administered irradiation at a total dose of 36 Gy to the whole bladder and part of tumour; nevertheless, radiotherapy was discontinued at a dose of 26 Gy because of the fact that she developed pollakisuria. She had pelvic magnetic resonance imaging and pathological examination of the urinary bladder after radiotherapy and these showed that the lymphoma was in complete remission; however, she received rituximab therapy at a dose of 375 mg/m^2^/week, 8 times additionally, because of the reduced radiotherapy. The patient had remained in complete remission for 14 months at the time of the report of her case.

Raderer et al. [11] stated that (a) various chemotherapeutic agents as well as the anti-CD20 antibody rituximab (R) had been tested in patients with mucosa-associated lymphoid tissue (MALT) lymphoma; however, no standard chemotherapeutic regimen had emerged so far; (b) judging from the data obtained in various types of lymphoma, the activity of R appears to be enhanced by combination with chemotherapy; (c) as no data on this topic exist for MALT lymphoma, they had retrospectively analysed their experience with R plus cyclophosphamide, doxorubicin/mitoxantrone, vincristine, and prednisone (R-CHOP/R-CNOP) in patients with relapsed MALT lymphoma. Raderer et al. [11] identified a total of 26 patients; 15 of these patients were administered R-CHOP while 11 patients were given R-CNOP due to age greater than 65 years or preexisting cardiac conditions. Cycles were repeated every 21 days, and restaging was performed after 4 cycles of therapy. In cases of complete remission, 2 further cycles were administered for consolidation while the patients who were achieving partial remission or stable disease after restaging were given 4 further courses. Raderer et al. [11] reported the following.

- A total of 170 cycles were administered to their patients (median 6, range 2 to 8).
- Twenty of the 26 patients (77%) achieved a complete remission and 6 (23%) a partial remission.
- Toxicities were mainly haematological, with WHO grade III/IV leukocytopenia occurring in 5 patients.
- After a median followup of 19 months (range 10 months to 45 months), all patients were alive: 22 were in ongoing remission, while 4 had relapsed between 12 and 19 months after treatment.

Raderer et al. [11] concluded that their data demonstrated a high activity of R-CHOP/R-CNOP in relapsing MALT lymphoma irrespective of prior therapy.

Finally, Tables 3 and 4 have been provided to summarize the reported experiences of a number of authors regarding the management of various types of lymphomas of the urinary bladder.

## 4. Conclusions 

Lymphoma of the urinary bladder may be either primary or secondary lymphoma.

Diagnosis of lymphoma of the urinary bladder is based upon the characteristic morphology of the bladder lesion which has been resected or biopsied and this must be supported by immunohistochemical analysis.

Lymphoma of the urinary bladder is a rare lesion.

Radiotherapy and chemotherapy are useful and effective in the treatment of lymphoma of the urinary bladder.

## Figures and Tables

**Table 1:** Clinical Summary of the four cases.

Case number	Age, years/sex	Presentation	Stage	Treatment	Follow-up years
1	64, female	Hematuria and frequency	IAE	Radiation	13
2	69, female	Frequency and urgency	IAE	Radiation	5
3	72, female	Hematuria and nocturia	IAE	Radiation	3
4	62, male	Hematuria and urgency	IAE	Radiation	2

Reproduced from [4] with permission of the Editor-in-Chief of Archives of Pathology and Laboratory Medicine on behalf of the editorial team of the journal and the American Association of Pathology.

**Table 2:** Immunohistochemical and polymerase chain reaction (PCR) findings*.

Case number	CD45	CD20	CD45RO	CD5	CD10	CD43	K & L	PCR
1	+	+	I	ND	ND	−	I	Monoclonal
2	+	+	−	−	−	−	LLCR	Monoclonal
3	+	+	−	−	−	−	KLCR	I
4	+	+	−	−	−	F+	K:CR	Monoclonal

*Plus sign indicates positive reaction; I: inconclusive; minus sign: negative reaction; ND: not done; F1: focally positive; LLCR: l-light chain restriction; and KLCR: k-light chain restriction.

Reproduced from [4] with permission of the Editor-in-Chief of Archives of Pathology and Laboratory Medicine on behalf of the editorial team of the journal and the American Association of Pathology.

**Table 3:** Some of the reported cases of lymphoma of the urinary bladder mainly primary with other cases of paraurethral lymphoma, their management and outcome.

References of cases	Treatment types	Follow-up duration	Complete remission	Partial remission	No response	Total sex/age histology
Raderer et al. [11]	RCHOP or RCNOP regime	19 months mean Range 10–45	20 (77%)	6 (23%)	0	26 patients with MALT lymphoma of bladder

Terasaki et al. [12]	Radiotherapy Gy 26 and Rituximab chemotherapy after remission	14 months	1 patient			1 female aged 64 years with MALT lymphoma of bladder

Takahara et al. [13]	TURBT and Radiotherapy 40 Gy in 20 fractions	3 monthly intervals to, duration is not available to author	1 patient			1 female aged 85 years with extranodal marginal zone B-cell lymphoma

Kakuta et al. [14]	Rituximab in combination with CHOPP chemotherapy after transurethral biopsy	Duration is not available to author	1 patient			1 female aged 84 years with extranodal marginal zone B-cell lymphoma of bladder

Siegel and Napoli [15]	Extensive resection	Duration is not stated	Alive, but outcome with regard to response is not available to author	Alive, but outcome with regard to response is not available to author		1 elderly female with B-cell lymphoma of dome of bladder with signet ring cell component

Hayashi et al. [16]	3 courses of R CHOPP chemotherapy	Duration is not available to author	1 patient			1 female age not available to author with DCBCL (primary diffuse large B-cell lymphoma of urinary bladder)

Abraham et al. [17]	Resectional biopsy and non-Hodgkin's lymphoma therapy	Duration is not stated	1			1 female aged 72 years with extranodal monocytoid B-cell lymphoma (MBCL) derived from marginal zone lymphocyte

Sundaram and Zhang [18]	Resection but details of further management not available	Details is not available to author				1 female aged 67 years with localized Epstein-Barr virus (EBV) positive B-cell lymphoproliferative disorder (LPD)/polymorphous B-cell lymphoma of the bladder

Oh and Zang [19]	Transurethral resection biopsy and two cycles of systemic cyclophosphamide, doxorubicin, vincristine, and prednisone (CHOPP) chemotherapy	Duration is not stated	1 patient with simultaneous restoration of urinary function			1 male aged 35 years with diffuse large B-cell lymphoma (non-Hodgkin's lymphoma)

Wang et al. [20]	TURBT and four cycles of CHOPP (cyclophosphamide, doxorubicin, vincristine, and prednisone) chemotherapy	12 months	1 with good response and remained in clinical remission for 12 months after treatment			1 male aged 45 years with T-cell lymphoma of urinary bladder

Mourad et al. [21]	Transurethral resection biopsy of lesion and CHOPP chemotherapy and he received cyclophosphamide, doxorubicin, vincristine, and prednisone	Duration not available to author: appeared case was reported earlier without details of long-term follow-up	Response not available			1 male aged 52 years who had shistosomiasis and found to have T-cell lymphoma of urinary bladder which Mourad et al. [21] felt was induced by shistosomiasis

Ando et al. [22]	Transurethral resection of bladder tumour only	3 years	1			1 female aged 77 years with primary low-grade B-cell lymphoma of the MALT type

Simpson et al. [23]	Details not available to author	Details are not available to author	Details not available to author	—	—	1 female with T-cell primary lymphoma of bladder and urethra

Mearini et al. [24]	Transurethral resection of bladder tumour (Burkitt's lymphoma) plus subsequent antiretroviral treatment with stavudine (40 mg twice daily), lamivudine (150 mg twice daily), and nelfinavir (750 mg 3 times daily), as well as antitumour polychemotherapy (4 cycles of cyclophosphamide, vincristine, doxorubicin, and dexamethasone, alternated with 4 cycles of methotrexate and cytarabine)	8 months of followup	Complete resolution and biopsy of small mucosal lesion at site of previous tumour 8 months later only showed fibrous tissue on immunohistochemical and histological examination			27-year-old man with Burkitt's lymphoma

Tsiriopoulos et al. [25]	Cystectomy and ileal conduit after failure of conservative treatment for presumed interstitial cystitis	Details are not available to author	Details are not available to author	Details are not available to author	Details are not available to author	75-year-old patient with past history of chronic lymphatic leukaemia histology of bladder showed primary splenic marginal zone lymphoma simulating interstitial cystitis

Downs et al. [26]	Details are not available to author	Details are not available to author	Details are not available to author	Details are not available to author	Details are not available to author	They concluded that primary lymphoma of the bladder has a good prognosis and responds to a variety of therapeutic modalities

Simpson et al. [27]	3 cases	7 years. 39 months. Details are not available to author	Alive and free of tumour. Died after 39 months. Details are not available to author			A 70-year-old man with low grade type A 67-year-old woman with intermediate-grade type 76-year-old woman with lymphoma in the urethra

Isaacson and Wright [28]	2 cases, details are not available to author	Details are not available to author	Details are not available to author	Details are not available to author	Details are not available to author	Details are not available to author

Ohsawa et al. [10]	3 cases, details are not available to author	Details are not available to author	Details are not available to author	Details are not available to author	Details are not available to author	Details are not available to author

Sönmezer et al. [9]	Transurethral biopsy, partial cystectomy, total hysterectomy, bilateral oophorectomy, and four courses of CHOP regimen (cyclophosphamide, vincristine, doxorubicin, and prednisolone)	6 years	Alive and well with no local recurrence of distant metastasis			1 female aged 50 years with high-grade B-cell lymphoma

Kuhara et al. [8]		Details of duration of followup are not available to author	Outcome is not available to author	Outcome is not available to author		Diffuse B-cell lymphoma of medium-sized cell

Sufrin et al. [7]	13% of 599 patients with malignant lymphoma had secondary bladder involvement and were treated with local radiotherapy	1952 to 1972	Good response			13% of 599 patients with secondary bladder lymphoma (details of the various types are not available to author)

Cohen et al. [6]	Details of case are not available to author	Details of case are not available to author	Details of case are not available to author	Details of case are not available to author	Details of case are not available to author	1 case of primary B-cell lymphoma of bladder

Zukerberg et al. [5]	5 cases (diagnosis of malignant lymphoma was excluded in 1 leaving 4 as lymphoma of T-cell type Of 2 muscle invasive tumours, 2 cases were too recent to have followup	4 Too recent for followup	1 alive with no tumour after 4 years following radiotherapy. and chemotherapy. Details are not available to author			

Al-Maghrabi et al. [4]	Radiotherapy (35 Gy)	13 years, 5 years, 3 years, 2 years, respectively	Alive no recurrence. Alive no recurrence. Alive no recurrence. Alive with no disease			64-year-old female,stage IAE 69-year-old female, low-grade MALT lymphoma, stage IAE 72-year-old female, low-grade lymphoma of MALT type-stage IAE 62-years-old male, B-cell malignant lymphoma of MALT type-stage IAE

Mantzarides et al. [29]	Details of treatment are not available to author	Further details are not available to author	Further details are not available to author	Further details are not available to author	Further details are not available to author	82-year-old female with primary diffuse large B-cell lymphoma of the bladder wall

Evans and Moore [30]	Transurethral biopsy of bladder tumour and she received a course of R-CHOP (cyclophosphamide, doxorubicin, vincristine, prednisolone, and rituximab) chemotherapy	4 months		CT scan showed regression of lesion and symptomatic improvement		64-year-old female with histologically proven diffuse large B-Cell non-Hodgkin's lymphoma (primary)

Arda et al. [31]	Open biopsy; she refused surgical operation and was referred to oncologist for chemotherapy	Further details are not available to author	Further details are not available to author	Further details are not available to author	Further details are not available to author	54-year-old female had open biopsy proven to be malignant non-Hodgkin's lymphoma

Aceñero, et al. [32]	Details are not available to author	Details are not available to author	Details are not available to author	Details are not available to author	Details are not available to author	3 cases of primary malignant lymphoma of urinary bladder (2 of high grade) of MALT type

Jacobs and Symington [33]	Cystectomy	3 years	Alive and well with no recurrence of locally or distant metastasis			61-year-old woman with primary lymphoma of urinary bladder

Diaz-Peromingo et al. [34]	TUR biopsies and CNOP (cyclophosphamide, mitoxantrone, vincristine, and prednisolone) and monoclonal antibodies anti-CD20	Short period of follow-up case reported shortly after initial treatment	Good initial response			79-year-old man tumour B-cell lymphoma (non-Hodgkin's) which was initially thought to be primary; however, PER scan confirmed that it was a secondary bladder lymphoma

Rijo et al. [35]	Open per vaginal partial excision of paraurerethal lesion. extending to the trigone of the bladder (this was a paraurethal lesion not a bladder lesion). Six cycles of immunochemotherapy: anti-CD20 (Rituximab) combined with chemotherapy (high doses of methotrexate and cytarabine with conventional cystostatics and prophylactic administration of G-CSF after chemotherapy cycles). After the completion of the third cycle of treatment, the patient achieved near-complete remission as well as a nearly complete regression of the paraurethral tumour and the lesion of the 5th lumbar vertebra. Haematological grade 2 toxicity and gastrointestinal grade 1 toxicity were reported	9 months	1			1 female aged 27 years

Hatano et al. [36]	Transurethral resection of bladder tumour and left total nephroureterectomy; histology adenocarcinoma G2pT2 in renal pelvis and MALT-type lymphoma of bladder; radiotherapy 36 Gy to bladder	14 months	Alive with no evidence of recurrence			84-year-old with MALT-type lymphoma of bladder and adenocarcinoma of left renal pelvis

**Table 4:** List of some of the reported studies on lymphoma of the urinary bladder with outcome.

References	Types and numbers of lymphomas of the urinary bladder	Types of management	Duration of followup	Outcome	Total
Bates et al. [2]	**6 cases of primary lymphoma** 3 extranodal marginal zone lymphoma of MALT type 3 diffuse B-cell lymphomas	Various details are not available 66 years/female, large bladder mass-low-grade MALT-type lymphoma T3	1 year	Indolent course with good prognosis Alive	1
		79 years/female Low-grade MALT-type	No followup	Unknown	1
		59 years female T2-T3 low grade MALT type		Alive with disease	1
		84 years/female, T3 diffuse large B-cell lymphoma	3 years 6 months	Died of disease after 6 months	
		67/years male, Solid tumour diffuse large B-cell lymphoma, had radiotherapy and chemotherapy	16 years	Alive with disease after 16 years	
		80 years/female Diffuse large B-cell lymphoma, had radiotherapy	3 years 8 months	Alive and well after 3 years 8 months	
	**5 cases of secondary lymphoma** 4 diffuse large B-cell lymphoma 1 nodular sclerosis non-Hodgkin's disease	Various details are not available to author	4 patients had followup up to 13 months	4 patients died within 13 months of followup	
	65-year-old male laparotomy showed mass involving ileum and generalised lymphadenopathy. Diffuse large B-cell lymphoma secondary to systemic follicular lymphoma		13 months	Died of disease after 13 months Died of disease after 10 months	
	41 years/male Diffuse large B-cell lymphoma had caecal mass and abdominal lymph adenopathy biopsy showed malignant lymphoma had radiotherapy and chemotherapy	Radiotherapy and chemotherapy	10 months		
	32 years/male, necropsy showed abdominal mass and lymphadenopathy. Diffuse large B-cell lymphoma		Died of disease. No followup	Died of disease. Died of disease after 1 month	
	76/female, mass in lower abdomen, swollen left leg, lymphadenopathy in left groin, and right axilla. Diffuse large B-cell lymphoma 81-year-old, female known Hodgkin's		1 month No followup	No follow-up	
	Disease developed to nodular sclerosis Hodgkin's disease of bladder				

Kempton et al. [3]	Primary B-cell MALT-type lymphoma of bladder in 6 patients Nonlocalized lymphoma (17 cases) 1 low-grade lymphoma of MALT type 12 large cell lymphoma 4 follicle centre lymphoma Secondary lymphoma occurred in 13 patients (2 with low-grade lymphoma of the MALT type; 1 with follicle centre lymphoma; 1 with mantle cell lymphoma; 9 with diffuse large B-cell lymphomas	Various	1940 to 1996	Complete remission. No patient died and no patient developed recurrent disease. Excluding two patients who died postoperatively, median survival was 9 years; 6 patients died of lymphoma in the follow-up group Median survival was 0.6 years	6 patients

Al-Maghrabi et al. [4]	4 cases of primary lymphoma (they had B-cell; centrocyte-like cells plasmacytoid or both)	All patients were treated by radiotherapy	2 to 13 years	Good prognosis (all the four with no recurrence and alive)	4 patients
